# Values of magnetic Resonance imaging and Cerebrospinal fluid analysis in the diagnosis of Central Nervous System associated infectious diseases

**DOI:** 10.12669/pjms.335.13083

**Published:** 2017

**Authors:** Dongfeng Zhang

**Affiliations:** 1 Dongfeng Zhang, Neurology Department, Henan Provincial People’s Hospital, Henan, 450000, China

**Keywords:** Central nervous system infection, Cerebrospinal fluid analysis, Magnetic resonance imaging

## Abstract

**Objective:**

To discuss the roles of magnetic resonance imaging (MRI) and cerebrospinal fluid analysis in the identification of central nervous system associated infection and provide a reference for the diagnosis and treatment of central nervous system associated infectious diseases.

**Methods:**

Seventy-six patients who developed central nervous system infection and were admitted into the Henan People’s Hospital between June 2014 and October 2015 were randomly selected as an observation group. Patients in the observation group were subdivided according to purulent meningitis, cryptococcal meningitis, viral meningitis and tubercular meningitis. Moreover, 35 headache patients who were admitted in the same period were selected as a control group. The MRI results and cerebrospinal fluid examination indicators were compared between the two groups.

**Results:**

MRI results suggested that the positive rate of the observation group was 96.05% (73/76), much higher than 8.57% in the control group (3/35), and the difference had statistical significance (P<0.05). The analysis results of cerebrospinal fluid demonstrated that the concentration of lactate dehydrogenase (LDH) in the cerebrospinal fluid of the patients with tubercular meningitis was the highest, the concentration of creatine kinase (CK) in the cerebrospinal fluid of the patients with purulent meningitis was the highest, and the concentration of lactic acid (LA) in the cerebrospinal fluid of the patients with tubercular meningitis and purulent meningitis was higher than that of the other patients; the differences were statistically significant (P<0.05). The analysis on the diagnostic efficacy of MRI in combination with cerebrospinal fluid analysis suggested that the sensitivity of the diagnostic scheme was high in diagnosing meningitis except purulent meningitis, and the specificity and accuracy of the scheme was high in diagnosing meningitis except cryptococcal meningitis.

**Conclusion:**

MRI in combination with cerebrospinal fluid analysis is effective in diagnosing central nervous system associated infectious diseases. It can also effectively identify the types of infection besides improving accuracy, which provides an important reference for clinical treatment.

## INTRODUCTION

Central nervous system infection (CNSI) refers to a series of commonly seen diseases including viral meningitis, purulent meningitis, tubercular meningitis and cryptococcal meningitis in the nervous system which are induced by invasion of pathogens such as fungus, spirochete, tubercle bacillus, bacteria and virus in spinal cord, brain parenchyma, vessels or envelope.^1^

CNSI featured by high disability rate and fatality rate, fast deterioration and acute onset can produce severe threatens to the lives of patients. Therefore, early diagnosis of CNSI is the key for the improvement of treatment effect.^2^,^3^

Viral meningitis, purulent meningitis, cryptococcal meningitis and tubercular meningitis manifests few typical features, and pathogenic results are difficult to be obtained in the early stage. Currently, the disease condition of patients with CNSI is usually evaluated based on history of disease, symptoms and cerebrospinal fluid detection results; however, the laboratory examination results and clinical manifestations of some patients are not typical, which severely disturbs the early diagnosis of CNSI and affect prognosis.^4^ In recent years, magnetic resonance imaging (MRI) and cerebrospinal fluid have been important examination approaches.^5^ The information provided by MRI is not more than many other imaging technologies, but also is different from that provided by the existing imaging technologies. Therefore, it suggests a huge potential superiority in the diagnosis of diseases.^6^ MRI can provide the images of the cross section, sagittal plane, coronal plane and various oblique planes, without causing artefacts like computed tomography (CT); moreover, it has no adverse effect on human body as imaging agents are not used and there is no ionizing radiation. Currently, MRI has been applied in the diagnosis of diseases in different systems of the whole body, especially the brain, spinal cord, cardiac great vessels, joint bones, soft tissues and pelvic cavity. Cerebrospinal fluid in normal human body has certain chemical components and pressure, which can maintain the relative stability of intracranial pressure. When there are central nervous system diseases, pathological changes will produce in the central nervous system and the metabolism of nervous cells will be disordered, which can change the property and components of cerebrospinal fluid. If the circulation path of cerebrospinal fluid is inhibited, intracranial pressure will increase. Therefore, the detection of cerebrospinal fluid is one of the important auxiliary diagnostic approaches for central nervous system impairment. Both MRI and cerebrospinal fluid can detect pathological changes in human body, which makes contributions to the prevention of diseases. Hence exploring MRI in combination with detection of cerebrospinal fluid has clinical values in diagnosing and identifying central nervous infection.

This study made a joint detection using MRI and biomechanical analysis on lactic dehydrogenase, lactic acid and creatine kinase in cerebrospinal fluid, aiming to investigate the identification and diagnosis of infectious diseases in central nervous system.

## METHODS

Seventy-six patients who developed CNSI and were admitted to the Henan People’s Hospital between June 2014 and October 2015 were randomly selected as the research subjects. They all obtained definite diagnosis through clinical examination. In the examination of cerebrospinal fluid, purulent meningitis patients with positive pathogenic bacteria, tubercular meningitis patients with positive tuberculin, viral meningitis patients with positive specific viral Ribose Nucleic Acid (RNA) and cryptococcal meningitis patients with Cryptococcus and moreover patients who signed informed consent were included. Those who suffered from severe diseases in organs such as the heart, liver and kidney and had incomplete basic data were excluded. In the observation group, there were 44 males and 32 females, with an average age of 52.61±10.34 years (22 to 78 years); the clinical pathological results confirmed 24 cases of tubercular meningitis, 21 cases of viral meningitis, 14 cases of purulent meningitis and 17 cases of cryptococcal meningitis. Moreover, 35 patients who received cerebrospinal fluid examination because of headache in the same period were selected and set as a control group. In the control group, there were 21 males and 14 females, with an average age of (51.13±10.18) years (23 to 76 years). The differences of general data between the two groups had no statistical significance (P>0.05); therefore, the results of the two groups were comparable.

A HD1.5T MR scanner produced by GE, America was used in MRI. Some patients were examined by enhancement scanning. Patients in the observation group underwent MRI twice or thrice, seven days after admission and seven days before discharge; some patients were re-examined once during hospitalization. Patients in the control group underwent MRI for more than one time. The examination results of the two groups were compared. It was determined as positive if there were areas with limited T1 and high-signal T2 weighted imaging (T2WI), central liquidation, multilocular isolation and focal abscess.

Lactate dehydrogenase (LDH), creatine kinase (CK) and lactic acid (LA) detection kits were used in the biochemical examination of cerebrospinal fluid associated indicators. AU 640 fully automatic biochemical analyser was also used. The use of all the instruments and reagents followed corresponding instructions. Cerebrospinal fluid was extracted after lumbar puncture. Purulent meningitis is induced by purulent bacterial infection, manifesting as turbid cerebrospinal fluid with a large amount of cells especially white blood cells, increased protein level and low sugar and chloride content. Tubercular meningitis is a non-suppurative inflammation induced by tubercle bacillus, manifesting as colorless and transparent or light yellow cerebrospinal fluid with significantly increased lymphocyte, increased proteins and decreased sugar and chloride. Viral meningitis is a leptomeningeal inflammatory response induced by viral infection, manifesting as colorless and transparent cerebrospinal fluid with normal or increased pressure, normal sugar and chloride content and slightly increased proteins.

### Statistical analysis

Data were processed using SPSS ver. 21.0. Categorical data were processed using Chi-square test. Measurement data were expressed as mean ± standard deviation (SD) and processed by t test. Difference was considered as statistically significant if P<0.05.

## RESULTS

### The MRI results of the two groups

**Fig.1:**
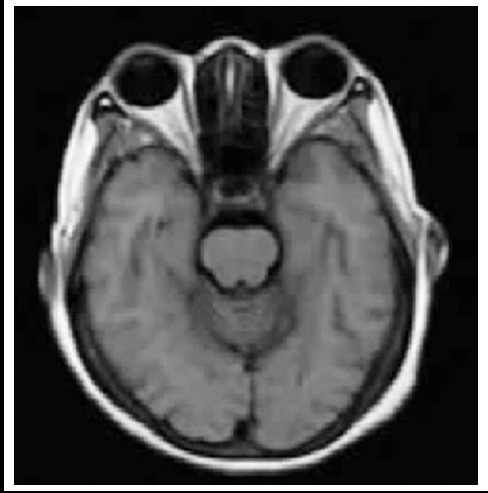
MRI plain scan images of viral meningitis (mainly menifested as multiple and irregular lesions with abnormal long-T1 and T2 signals)

The MRI results demonstrated that, the positive rate of the observation group was 96.05%; the positive rate of the tubercular meningitis group and the cryptococcal meningitis group was both 100%; the positive rate of the viral meningitis group (Fig.1) and the purulent meningitis group was 90.48% and 92.86% respectively. The positive rate of the control group was 8.57%. The difference of the positive rate between the two groups had statistical significance (X^2^=10.317, P<0.05; Table-I).

**Table-I:** The comparison of the positive rate of MRI between the two groups.

*Group*	*N*	*Number of positive cases*	*Positive rate (%)*
Observation group	76	73	96.05
Tubercular meningitis group	24	24	100
Purulent meningitis group	14	13	92.86
Viral meningitis group	21	19	90.48
Cryptococcal meningitis group	17	17	100
Control group	35	3	8.57

### The examination results of LDH, CK and LA between the two groups

The examination results of the cerebrospinal fluid demonstrated that, the LDH concentration of the tubercular meningitis group was the highest, the CK concentration of the purulent meningitis group was the highest, and the LA concentration of the tubercular meningitis group and purulent meningitis group was higher than the other two groups. The comparison of the LDH, CK and LA concentration in the cerebrospinal fluid between the groups suggested statistically significant differences (P<0.05; Table-II).

**Table-II:** The comparison of the cerebrospinal fluid examination results between the two groups.

*Group*		*N*	*LDH (U/L)*	*CK (U/L)*	*LA (U/L)*
Observation group (N=76)	Tubercular meningitis group	24	86.35±10.18	10.47±4.85	55.91±7.84
	Purulent meningitis group	14	72.47±15.29	1.72±1.66	12.61±2.93
	Viral meningitis group	21	16.88±8.71	0.98±0.72	10.06±8.24
	Cryptococcal meningitis group	17	15.89±7.63	16.93±3.91	53.34±8.41
Control group		35	16.25±8.01	0.88±0.67	11.77±2.23
F value		7.849	10.652	9.248	
P value		<0.05	<0.05	<0.05	

### The diagnostic efficacy of MRI in combination with cerebrospinal fluid analysis in diagnosing CNSI

The sensitivity of MRI in combination with cerebrospinal fluid analysis in diagnosing tubercular meningitis, viral meningitis and cryptococcal meningitis was higher than that in diagnosing purulent meningitis. The specificity and accuracy of MRI in combination with cerebrospinal fluid analysis in diagnosing viral meningitis, purulent meningitis and tubercular meningitis was much higher than that in diagnosing cryptococcal meningitis (Table-III).

**Table-III:** The diagnostic efficacy of MRI in combination with cerebrospinal fluid analysis in diagnosing CNSI (%).

*Indicator*	*Tubercular meningitis*	*Viral meningitis*	*Cryptococcal meningitis*	*Purulent meningitis*
Sensitivity	62.46	68.71	64.56	24.75
Specificity	80.35	76.96	57.48	75.36
Accuracy	78.22	71.47	56.07	65.11

## DISCUSSION

MRI, a new medical technology of CT, has been extensively applied in the diagnosis in various clinical departments because of many advantages.^7^ Firstly, multidimensional image data can be obtained through MRI, which can provide more intuitive and comprehensive information about human body. Secondly, it will not produce radiation damages on human body, suggesting high safety. Finally, it can clearly display soft tissue structure. Therefore, MRI can effectively identify the central nervous system and accurately detect lesions.^8^ Cheng S. et al. found that MRI and computed tomography (CT) could be used to accurately and intuitively observe lesions in the brain,^6^ featured by simple operation and high efficiency.^9^ Spudich S. et al. found that MRI has considerable advantages in diagnosing the correlation between tissues around lesions and the internal structure as well as the size,8 number and distribution scope of lesions, but the specificity was low.^10^ In this study, the positive rate of MRI was 96.05% in the observation group. But someone put forward that MRI consumed much time in identifying CNSI and was not applicable to critically ill patient.^11^ Basal cistern or even lateral fissure enhancement, hydrocephalus and basal ganglia lacunar lesions displayed by MRI are considered as three characteristics of tubercular meningitis. As to purulent meningitis, mater enhancement is not common in enhancement scanning, and only few cases were observed with inorganic adhesion and communicating hydrocephalus because of effusion. Cryptococcal meningitis also manifests meningitis symptoms in clinics; however, cerebral base and mater enhancement and hydrocephalus are usually not obvious or only non-enhanced colloid pseudocyst or slightly enhanced cryptococcal tumors are displayed, and sometimes expanded peripheral space of special Virchow vessels in cerebral base can be seen.^12^

Cerebrospinal fluid examination has been one of the methods which have been extensively applied for diagnosing CNSI and also the golden standard for the acquisition of etiological basis.^13^ The application of etiological examination in the early diagnosis is easily affected by the external factors and moreover low-efficient.^14^ Cerebrospinal fluid analysis aims at objectively evaluating the content of enzymes in cerebrospinal fluid. Generally, the cerebrospinal fluid of normal people contains more than 20 kinds of enzymes. When some diseases occur to the nervous system, the content of enzymes in cerebrospinal fluid of patients with CNSI will increase immediately due to the failure of blood brain barrier function. Therefore, the changes of the content of enzymes in crebrospinal fluid reflect the severity of cerebral injury to some extent.^15^,^16^ This study further evaluated the injury of blood brain barrier and brain tissues through detecting the concentration of LDH, CK and LA in cerebrospinal fluid. LDH is distributed extensively in various tissues across human body. The increased LDH content in cerebrospinal fluid indicates a high probability of injuries in the central nervous system. CK is cytosolic enzyme which is extensively distributed in cytoplasm and mitochondria. The content of CK can reflect the damage extent of cerebral tissues and the changes of blood brain barrier. LA is the final product of anaerobic glycolysis of body metabolism, and its content can reflect the demand-supply equilibrium of oxygen in brain tissue.

The research results demonstrated that, the sensitivity of MRI in combination with cerebrospinal fluid analysis cerebrospinal fluid analysis was sensitive in identifying tubercular meningitis, viral meningitis and cryptococcal meningitis, but the accuracy and specificity of the diagnostic method were low in identifying cryptococcal meningitis, indicating the method had high application values in diagnosing CNSI.

### Limitations of the study

The samples included in this study were selected from the same hospital. Therefore regional difference could not be avoided in the results obtained. Precise verification involving multiple regions and large sample size is needed in the future.

## CONCLUSION

In conclusion, MRI in combination with cerebrospinal fluid analysis plays a crucial role in the identification of CNSI, but it suggests no specific performance in the comparison of imaging examination results, history of diseases and other manifestations. MRI in combination with cerebrospinal fluid analysis can improve the diagnosis rate.
